# Early Discharge from the Emergency Department Based on Soluble Urokinase Plasminogen Activator Receptor (suPAR) Levels: A TRIAGE III Substudy

**DOI:** 10.1155/2019/3403549

**Published:** 2019-05-19

**Authors:** Martin Schultz, Line J. H. Rasmussen, Thomas Høi-Hansen, Erik Kjøller, Birgitte N. Jensen, Morten N. Lind, Lisbet Ravn, Thomas Kallemose, Theis Lange, Lars Køber, Lars S. Rasmussen, Jesper Eugen-Olsen, Kasper K. Iversen

**Affiliations:** ^**1**^ Department of Cardiology, Herlev and Gentofte Hospital, University of Copenhagen, Ringvej 75, 2730 Herlev, Denmark; ^2^Department of Internal Medicine and Geriatrics, Herlev and Gentofte Hospital, University of Copenhagen, Ringvej 75, 2730 Herlev, Denmark; ^3^Clinical Research Centre, Amager and Hvidovre Hospital, University of Copenhagen, Kettegård Alle 30, 2650 Hvidovre, Denmark; ^4^Department of Emergency Medicine, Bispebjerg Hospital, University of Copenhagen, Bispebjerg Bakke 23, 2400 Copenhagen, Denmark; ^5^Department of Emergency Medicine, Herlev and Gentofte Hospital, University of Copenhagen, Herlev Ringvej 75, 2730 Herlev, Denmark; ^6^Department of Public Health, University of Copenhagen, Section of Biostatistics, Øster Farimagsgade 5, 1014 Copenhagen, Denmark; ^7^Center for Statistical Science, Peking University, No. 5 Yiheyuan Road Haidian District, Beijing 100871, China; ^8^Department of Cardiology, Rigshospitalet, University of Copenhagen, Blegdamsvej 9, 2100 Copenhagen, Denmark; ^9^Department of Anaesthesia, Centre of Head and Orthopaedics, Rigshospitalet, University of Copenhagen, Blegdamsvej 9, 2100 Copenhagen, Denmark

## Abstract

**Objective:**

Using biomarkers for early and accurate identification of patients at low risk of serious illness may improve the flow in the emergency department (ED) by classifying these patients as nonurgent or even suitable for discharge. A potential biomarker for this purpose is *soluble urokinase plasminogen activator receptor* (suPAR). We hypothesized that availability of suPAR might lead to a higher proportion of early discharges.

**Design:**

A substudy of the interventional TRIAGE III trial, comparing patients with a valid suPAR measurement at admission to those without. The primary endpoint was the proportion of patients discharged alive from the ED within 24 hours. Secondary outcomes were length of hospital stay, readmissions, and mortality within 30 days.

**Setting:**

EDs at two university hospitals in the Capital Region of Denmark.

**Participants:**

16,801 acutely admitted patients were included.

**Measurements and Main Results:**

The suPAR level was available in 7,905 patients (suPAR group), but not in 8,896 (control group). The proportion of patients who were discharged within 24 hours of admittance was significantly higher in the suPAR group compared to the control group (50.2% (3,966 patients) vs. 48.6% (4,317 patients), *P* = 0.04). Furthermore, the mean length of hospital stay in the suPAR group was significantly shorter compared to that in the control group (4.3 days (SD 7.4) vs. 4.6 days (SD 9.4), *P* = 0.04). In contrast, the readmission rate within 30 days was significantly higher in the suPAR group (10.6% (839 patients) vs. 8.8% (785 patients), *P* < 0.001). Among patients discharged within 24 hours, there was no significant difference in the readmission rate or mortality within 30 days. Readmission occurred in 8.5% (336 patients) vs. 7.7% (331 patients) (*P* = 0.18) and mortality in 1.3% (52 patients) vs. 1.8% (77 patients) (*P* = 0.08) for the suPAR group and control group, respectively.

**Conclusion:**

These post hoc analyses demonstrate that the availability of the prognostic biomarker suPAR was associated with a higher proportion of discharge within 24 hours and reduced length of stay, but more readmissions. In patients discharged within 24 hours, there was no difference in readmission or mortality.

**Trial Registration of the Main Trial:**

This trial is registered with NCT02643459.

## 1. Introduction

Early and accurate identification of patients at low risk of serious illness may improve the flow in the emergency department (ED) by classifying these patients as nonurgent or even suitable for discharge [1, 2]. This would allow for a better utilization of limited staff and resources and could potentially translate into improved patient outcomes. Previous research has suggested that blood-based prognostic biomarkers measured at admittance can be used for this purpose [3–9]. One of these prognostic biomarkers is *soluble urokinase plasminogen activator receptor* (suPAR), which was found in recent studies to be a strong and nonspecific marker of the presence and severity of a wide range of diseases and overall prognosis in ED patients, making suPAR suitable for risk stratification [3, 10–15].

The TRIAGE III trial investigated the effect of introducing suPAR in the EDs for improving early risk stratification and as a clinical decision tool [16, 17]. Despite the good abilities to discriminate between patients at high and low risk of mortality, we found no effect on all-cause mortality when suPAR was introduced to enhance risk stratification [17].

Here, we hypothesize that due to the nonspecific nature of suPAR, the biomarker might be most appropriate for identifying patients at very low risk. The aim of this post hoc substudy of the TRIAGE III trial was to investigate if availability of suPAR had an impact on discharge decisions leading to a higher proportion of early discharges within 24 hours from the ED.

## 2. Materials and Methods

### 2.1. Setting and Design

**Figure 1:**
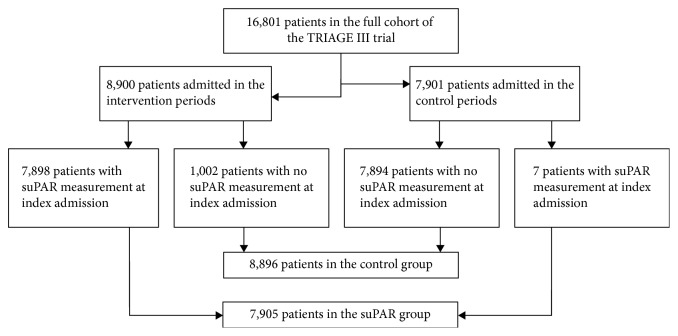
Flow diagram of the study population. The figure shows the TRIAGE III study population and the composition of the groups, with regard to the presence (suPAR group) and absence (control group) of suPAR in patients acutely admitted to two emergency departments (EDs) studied in this study.

In this post hoc substudy, we used the data on the same consecutively included and unselected population as in the TRIAGE III trial. As early discharge based on suPAR would require availability of the suPAR level, we compared patients that had a valid suPAR measurement to those without, regardless of whether patients arrived in interventional or control periods (Figure 1).

The protocol and primary results of the TRIAGE III trial have been published previously [16, 17]. In brief, TRIAGE III was a cluster-randomized interventional trial investigating the effect on mortality when suPAR was introduced in the ED. In interventional periods, suPAR measurement was available along with the routine blood tests at acute admission. Using the point-of-care equipment, “suPARnostic® Quick Triage”, the suPAR results were shown to the ED staff on monitors and in the electronic patient records within 2 hours of admission. Prior to the TRIAGE III trial, doctors at the EDs were informed about the prognostic ability of suPAR through oral presentations, a brief review on the published literature, and pocket cards. Two main advices were given to the staff: (1) if the suPAR level is elevated, the risk of death is high and the patient should receive a high level of attention and (2) if the suPAR level is low, the risk of life-threatening disease and death is low and early discharge of the patient should therefore be considered [16]. In control periods, suPAR was not measured.

### 2.2. Data

Patients included in the TRIAGE III trial were acutely admitted to one of the participating EDs. The first admission in the inclusion period was defined as their index admission, and only the index admission was included in the analyses. Data on blood tests were extracted from the electronic hospital database via the Department of Clinical Biochemistry. Routine blood tests included levels of albumin, creatinine, C-reactive protein (CRP), and haemoglobin. We acquired data on baseline characteristics, hospital admissions, and death from the Danish National Registries. All contacts at Danish hospitals, including dates of admission and discharge and diagnoses, are registered in the Danish National Patient Registry (DNPR), and the Danish Civil Registration System contains information on sex, date of birth, and vital status [18, 19]. The Charlson comorbidity index (Charlson score) was calculated using a modified SAS macro and based on all diagnoses in the DNPR that were registered two years prior to the index admission [20].

### 2.3. Outcomes

We calculated the proportion of patients discharged alive from the ED within 24 hours (early discharge), the length of hospital stay, and the number of readmissions within 30 days according to whether a valid suPAR level was available at admission. Furthermore, to assess safety in early discharge decisions associated with the suPAR level, we calculated 30-day all-cause mortality adjusted for differences in baseline variables along with 30-day readmission rates of patients discharged within 24 hours. Finally, we assessed the predictive ability of suPAR regarding 30-day mortality and readmissions in patients discharged within 24 hours.

### 2.4. Statistical Analysis

Continuous variables are presented as median and interquartile range (IQR), and categorical variables as number (*n*) and percentages (%). Baseline characteristics were compared using the chi-square test, Student's independent two-sample *t*-test, and Wilcoxon rank-sum test. Mortality was compared using logistic regression and Fisher's exact test. Furthermore, mortality was assessed in a multivariable logistic regression model adjusted for age, sex, hospital, haemoglobin, and albumin levels and finally in a model adjusted for all baseline variables, and results are presented as odds ratios (OR) with 95% confidence intervals (CI); in all analyses, the control group serves as the reference. Kaplan-Meier plots were used to illustrate survival, and the log-rank test was used to compare survival. Readmission rates were reported as proportions and compared using Fisher's exact test, additionally with a Cox model, where results are presented as hazard ratios (HR) with CI. The predictive ability of suPAR with regard to 30-day mortality was assessed with the area under the curve (AUC) for receiver operating characteristic (ROC) curves. Comparison of AUC between different models was done by the DeLong method [21].

*P* < 0.05 was considered statistically significant. Statistics was performed in R version 3.2.3 [22–24].

### 2.5. Ethics

The TRIAGE III trial was approved by the Danish Data Protection Agency (HGH-2015-042, I-Suite no. 04087) as well as the Danish Patient Safety Authority (Ref. no. 3-3013-1744/1). The trial was presented to the Regional Ethics Committee who decided that no formal approval was needed for the cluster-randomized TRIAGE III trial and that it could be conducted without the consent of the patients in accordance with the Danish law (Ref. no. FSP-15003590). No further permissions were required for this substudy.

## 3. Results

### 3.1. Trial Population

All data of the post hoc analyses were calculated on the basis of the TRIAGE III data set, which included 26,653 acute admissions of 16,801 unique patients. Patients were included in the TRIAGE III trial from January 11, 2016, to June 6, 2016. The suPAR level was available at the index admission in 7,905 patients (suPAR group), and no value was available in 8,896 (control group) (Figure 1 and Table S1). There were no missing data. Baseline characteristics were comparable between the groups; however, there was a significant difference in the range of the albumin and CRP levels.  Table S2 compares patients without a valid suPAR measurement arriving in the intervention period (*N* = 1,002) and patients arriving in the control period (*N* = 7,898). Apart from small but significant differences in the mean Charlson score (lower in the control group) and levels of CRP and albumin (higher in the control group), the two groups were comparable.

### 3.2. Outcomes in the Total Cohort

The mean age of the trial population was 64 years (IQR 45–77), and 8,864 (52.8%) were women. A total of 58 (0.3%) patients died within 24 hours, and 678 (4.0%) patients died within 30 days. The proportion of patients who were discharged within 24 hours of admittance was significantly higher in the suPAR group compared to the control group (50.2% (3,966 patients) vs. 48.6% (4,317 patients), absolute difference: 1.6% (95% CI: 0.08–3.12); *P* = 0.04). Baseline characteristics of patients discharged within 24 hours compared to those of patients with longer admissions are shown in Table S3. The mean length of stay in the ED was 8.4 hours (SD 6.5). The median suPAR level was significantly lower in patients who were discharged early: 3.5 ng/mL (IQR 2.6–4.8) vs. 4.9 ng/mL (IQR 3.5–7.2) (*P* < 0.001). Patients discharged within 24 hours were significantly younger and had a lower Charlson score, creatinine, and CRP levels as well as higher albumin and haemoglobin levels. Furthermore, the mean length of hospital stay in the suPAR group was significantly shorter during the index admission compared to that in the control group (4.3 days (SD 7.4) vs. 4.6 days (SD 9.4), difference in hours: 6.5 (95% CI: 0.2–12.7); *P* = 0.04). In contrast, the readmission rate within 30 days was significantly higher in the suPAR group (10.6% (839 patients) vs. 8.8% (785 patients), absolute difference: 1.8% (95% CI: 0.9–2.7); *P* < 0.001) (Table S1). Outcomes stratified according to hospitals are reported in Table S4.

### 3.3. Death following Early Discharge

**Figure 2:**
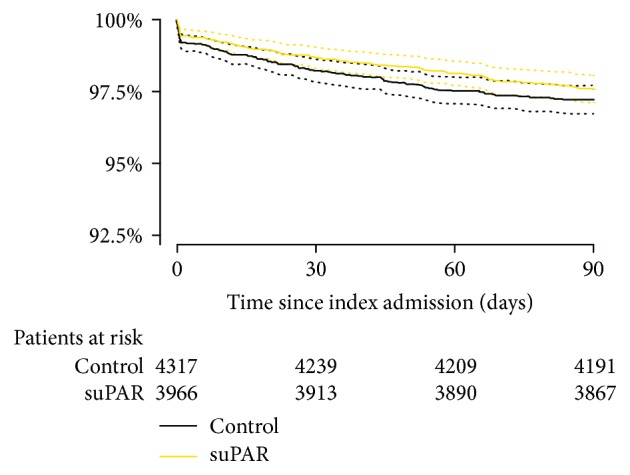
Kaplan-Meier plot. The figure displays the survival of patients discharged within 24 hours from emergency departments stratified by the presence (suPAR group) or absence (control group) of soluble urokinase plasminogen activator receptor at admission. Log-rank test: *P* = 0.3.

Baseline characteristics at the index admission of patients discharged alive within 24 hours from the ED stratified by the presence (suPAR group) or absence (control group) of suPAR are presented in Table 1. There were significant differences in the level of albumin and haemoglobin. Death within 30 days occurred in 52 patients (1.3%) in the suPAR group and in 77 patients (1.8%) in the control group. None of the logistic regression analyses revealed significant differences in mortality between groups: unadjusted (OR: 0.73, 95% CI: 0.51–1.04; *P* = 0.08) in the suPAR group compared to the control group, adjusted for age, sex, hospital, albumin, and haemoglobin (OR: 0.80, 95% CI: 0.54–1.19; *P* = 0.28), and fully adjusted model (OR: 0.74, 95 CI: 0.49–1.12; *P* = 0.16) (Table 2). Survival within 90 days following discharge is displayed in Figure 2.

### 3.4. Readmissions following Early Discharge

No significant difference was found in the readmission rate within 30 days between the two groups of patients discharged within 24 hours. Readmission within 30 days occurred in 336 patients (8.5%) vs. 331 patients (7.7%) (*P* = 0.18) for the suPAR group and control group, respectively. The Cox model with competing risks of death found no difference between the groups in readmission within 30 days: HR: 1.1 (95% CI: 0.95–1.29, *P* = 0.20).

### 3.5. Predictive Abilities of suPAR in Early Discharged Patients

**Figure 3:**
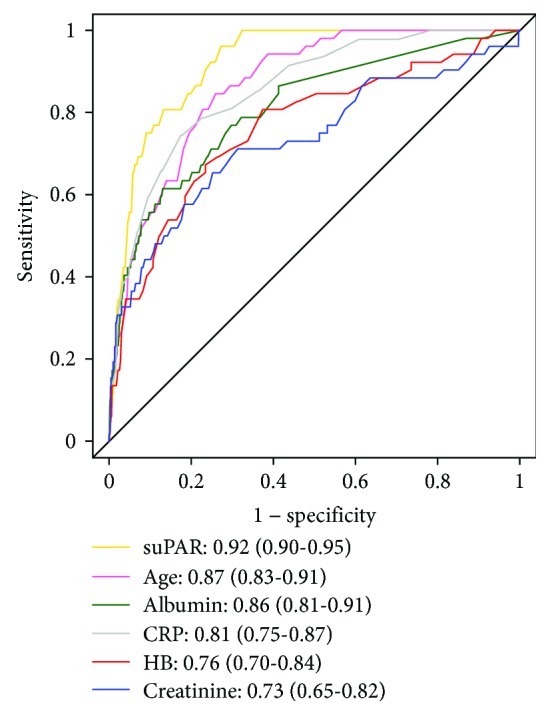
ROC curve for 30-day mortality. Receiver operating characteristic (ROC) curves displaying the predictive abilities of age, routine biomarkers, and soluble urokinase plasminogen activator receptor (suPAR) regarding 30-day all-cause mortality. Results were reported as the area under the curve with 95% confidence intervals.

In patients discharged within 24 hours, the suPAR level differed between survivors and nonsurvivors at 30 days (median 3.5 ng/mL (2.6–4.7) vs. 8.5 ng/mL (6.7–11.8), *P* < 0.001). No deaths occurred within 30 days in patients with a suPAR level below 4.3 ng/mL. The AUC for predicting 30-day mortality was 0.92 (95% CI 0.90–0.95). Stratifying suPAR levels on quartiles revealed a good discriminative power especially in the upper quartile compared to the lower quartiles (Figure S1). AUC comparison of age and analysed routine biomarkers (blood levels of albumin, creatinine, CRP, and haemoglobin) found age to be superior in predicting mortality compared to routine biomarkers at 30 days, only surpassed by suPAR (Figure 3 and Table 2).

A prediction model for 30-day mortality consisting of a combination of biomarkers (containing blood levels of albumin, creatinine, CRP, and haemoglobin) was significantly improved by adding suPAR (AUC 0.94 (95% CI 0.91–0.96), *P* = 0.006), and suPAR predicted 30-day mortality equally as good as the combined prediction model: AUC 0.92 (95% CI 0.90–0.95) and AUC 0.92 (95% CI 0.89–0.95), respectively (Figure S2). When using a combined outcome of readmission or death within 30 days, the combined biomarker model with an AUC of 0.92 (95% CI 0.89–0.95) was significantly improved when suPAR was added (AUC 0.94 (95% CI 0.91–0.96), *P* = 0.01).

## 4. Discussion

In this post hoc analysis study of a large cluster-randomized trial, we found a significantly higher proportion of patients discharged within 24 hours from the ED and a significantly lower mean length of stay in the suPAR group compared to the control group; however, these findings were accompanied by a significantly higher frequency of readmissions within 30 days in the suPAR group. In patients discharged within 24 hours, we observed no difference in all-cause mortality or readmissions within 30 days according to whether suPAR was available.

These analyses were aimed at evaluating the effect of using suPAR as a supportive tool when deciding on early discharge from the ED as well as the prognostic value of suPAR in predicting mortality. Our results indicate that a decision to discharge based on the suPAR level is safe and feasible in terms of the outcomes investigated. However, results of a biomarker should be considered alongside other clinical features in an overall assessment of the patient, as several acute conditions require immediate attention and treatment, regardless of the suPAR level. In this study, we observed that availability of suPAR in the ED improved flow parameters (length of stay and number of discharges), which potentially can reduce crowding, improve utilization of resources and patient outcomes, and lead to savings in the health care system. One potential drawback however is the increased readmissions. The results presented here also demonstrate superiority of suPAR as a prognostic biomarker compared to other investigated biomarkers, including a combined model of commonly used routine blood tests, in predicting short-term mortality. The ED staff was advised to consider early discharge when the suPAR level was low and if the other clinical findings did not contradict this. The high AUC (0.92) of suPAR when predicting 30-day mortality might reflect that the ED staff was able to perform a more accurate assessment of patients at risk of mortality, when they had the suPAR level available.

The prognostic abilities of suPAR have been studied before [3, 10–12], and the biomarker appears to be a potential candidate for stratifying patients according to risk of mortality and adverse events in emergency medicine [4, 5]. Previous studies have focused on high-risk patients (high suPAR) and to a lesser extent investigated the clinical impact of targeting patients with low suPAR levels. The nature of suPAR is highly nonspecific, and plasma levels are associated with a wide range of chronic conditions, cancer, and adverse events during hospitalization [3, 25–31]. Due to these properties, a low suPAR level may be used to identify patients with a good prognosis and at very low risk of short-term mortality [3, 17]. This ability could be valuable when assessing a larger number of patients in a short time at a busy ED. Improving patient flow by early discharge of low-risk patients, where admission might not be necessary, will potentially benefit both patients in need of hospital treatment and low-risk patients that can be discharged without being exposed to the risks of hospitalization.

This study has several limitations. The interpretation and generalizability of these results are limited as the study is based on post hoc analyses and was conducted at two hospitals in the same region. These explorative results must be confirmed using a prospective approach focusing on discharge. The TRIAGE III trial was originally designed to detect changes in overall 10-month mortality and thus overpowered in detecting differences in outcomes regarding early discharge or length of stay. It should be considered that the observed differences are small, and whether they can be considered clinically important depends on the setting. Furthermore, the analyses presented here are based on two groups consisting of patients with and without a suPAR measured at admission, regardless of the allocation in the original cluster randomization and not in accordance with the intention-to-treat principle, which might cause bias. This approach can be discussed, and we do not know exactly why suPAR was not always measured in the interventional period. There were small, but significant, differences in the Charlson score, albumin, and CRP levels, which we do not consider clinically important, and we found no differences in the baseline characteristics or in mortality between patients without a suPAR measurement in the intervention periods and patients included in the control periods. However, this approach might also be necessary to assess the impact of using suPAR in clinical practice, as ED doctors will not be able to guide interventions based on the biomarker level without the result. We chose to assess outcomes using the Danish registries as previous research has found that registry-based outcomes are similar to those obtained using adjudication committees [32]. Furthermore, when assessing new initiatives in risk stratification in the ED, there are several other important outcomes that should be assessed and reported (i.e., time to relevant treatment, crowding, intensive care admissions, and unexpected deterioration).

Although all outcomes presented here were included in the original analysis plan [33], the findings in this secondary study must be interpreted cautiously and be considered hypothesis-generating. Thus, it will be reasonable to conduct a future interventional study focusing on the negative predictive value of suPAR with clearly defined interventions, such as recommendation for rapid discharge below a predefined cut-off value with subsequent follow-up at outpatient clinics or by general practitioners.

## 5. Conclusion

These post hoc analyses demonstrate that the availability of the prognostic biomarker suPAR may lead to reduced length of stay and allow more discharges within 24 hours, however, the overall readmittance rate was increased. Patients who were discharged early, where suPAR was available, had no increased risk of mortality or readmission within 30 days compared to those being in the standard care. Usage of biomarker-based prognostic information for clinical decisions is a concept that still needs additional research before being implemented in routine practice but has potential to improve patient health.

## Figures and Tables

**Table 1:** Characteristic of patients discharged from the emergency department within 24 hours based on the presence (suPAR group) or absence (control group) of suPAR at the index admission.

	suPAR (*N* = 3,966)	Control (*N* = 4,317)
Hospital, no. of patients (%)		
Bispebjerg Hospital	1,222 (30.8)	1,744 (40.4)
Herlev Hospital	2,744 (69.2)	2,573 (59.6)
Patients		
Female sex—no. (%)	2,147 (54.1)	2,284 (52.9)
Age (years)—mean (SD)	53.6 (20.5)	53.5 (20.7)
Charlson score—mean (SD)	0.5 (1.2)	0.5 (1.2)
Blood levels of biomarkers, median (IQR)		
Albumin (g/L)	41 (38–44)	41 (37– 44)^∗^
Creatinine (*μ*mol/L)	72 (61–87)	73 (62–87)
CRP (mg/L)	3 (3–14)	3 (3–13)
Haemoglobin (mmol/L)	8.5 (7.9–9.2)	8.6 (7.9–9.2)^∗^
suPAR (ng/mL)	3.5 (2.6-4.8)	n.a.

CRP: C-reactive protein; IQR: interquartile range; n.a.: not available; SD: standard deviation; suPAR: soluble urokinase plasminogen activator receptor. ^∗^ *P* < 0.05.

**Table 2:** 30-day mortality of patients discharged within 24 hours. Groups were created based on the presence (suPAR group) or absence (control group) of suPAR at the index admission.

Mortality, no. patients (%)	
suPAR group	52 (1.3)
Control group	77 (1.8)
suPAR level at index admission (ng/mL)	
Alive, median (IQR)	3.5 (2.6–4.7)
Dead, median (IQR)	8.5 (6.7–11.8)^∗^
Logistic regression models, OR (95% CI)	
Unadjusted	0.73 (0.51–1.04)
Adjusted for age, sex, hospital, haemoglobin, and albumin level	0.80 (0.54–1.19)
Fully adjusted, all baseline variables	0.74 (0.49–1.12)
Area under the curve (95% CI)	
Age	0.87 (0.83–0.91)
Albumin	0.86 (0.81–0.91)
Creatinine	0.73 (0.65–0.82)
CRP	0.81 (0.75–0.80)
Haemoglobin	0.76 (0.70–0.84)
suPAR	0.92 (0.90–0.95)

CI: confidence interval; CRP: C-reactive protein; IQR: interquartile range; OR: odds ratio for the suPAR group compared to the control group; suPAR: soluble urokinase plasminogen activator receptor. ^∗^ *P* < 0.05.

## Data Availability

Individual data was collected for this study and can be made available in an anonymised form upon reasonable request. However, in accordance with the Danish law, this is conditioned by acceptance from the Danish Protection Agency. Interested investigators who propose to use the data that has been approved by an independent committee can contact the corresponding author and can receive deidentified individual participant data that underlie the results reported in this article. The study protocol and statistical analysis plan from the TRIAGE III trial will be available prior to submission by request. Data and documents are available 5 years following publication.

## Supplementary Materials

Supplementary Materials The file contains Tables S1, S2, S3, and S4 and Figures S1 and S2. These supplementing tables and figures provide a more in-depth understanding of the manuscript. Table S1: patient characteristics and outcomes. Groups were created based on whether suPAR was measured and reported at the index admission (suPAR group) or not (control group). Table S2: patient characteristics. Comparison of patients admitted in control periods and patients arriving in interventional periods, but who did not have a valid suPAR level at admission. Table S3: patient characteristics at index admission for patients discharged from the emergency department within 24 hours compared to patients with longer admissions. Table S4: patient characteristics and outcomes stratified according to hospital. Figure S1: survival of patients discharged within 24 hours from the emergency department stratified according to suPAR quartiles measured at index admission. Log-rank test: *P* < 0.001. Figure S2: ROC curve comparison of a biomarker model and suPAR. Receiver operating characteristic curves for 30-day all-cause mortality displaying predictive abilities of a biomarker model (including levels of albumin, creatinine, C-reactive protein, and haemoglobin), the biomarker soluble urokinase plasminogen activator receptor (suPAR), and the biomarker model combined with suPAR. No suPAR: a biomarker model without suPAR; suPAR included: a biomarker model including suPAR.
