# Supplementary material for: Early Discharge from the Emergency Department Based on Soluble Urokinase Plasminogen Activator Receptor (suPAR) Levels: A TRIAGE III Substudy
Source: Dis Markers. 2019 May 19;2019:3403549. doi: 10.1155/2019/3403549 (PMC6545801; doi:10.1155/2019/3403549)

SUPPLEMENTARY MATERIAL

Table S1. Patient characteristics and outcomes. Groups were created based on whether suPAR was measured and reported at the index admission (suPAR group) or not (control group).

Characteristic	suPAR group (N=7,905)	Control group (N=8,896)	P
**Hospital, no. of patients (%)**			
Bispebjerg Hospital	2,895 (36.6)	4,125 (46.4)	
Herlev Hospital	5,010 (63.4)	4,771 (53.6)	
**Patients**			
Female sex, no. (%)	4,173 (52.8)	4,691 (52.9)	0.95
Age, years, mean (SD)	60.5 (20.7)	60.7 (20.8)	0.47
Charlson score, mean (SD)	0.7 (1.5)	0.7 (1.5)	0.42
**Biomarker blood level, median (IQR)**			
Albumin (g/L)	39 (35–43)	39 (34–42)	<0.001*
Creatinine (μmol/L)	75 (62–94)	75 (63–93)	0.75
CRP (mg/L)	5 (3–41)	5 (3–40)	0.02*
Hemoglobin (mmol/L)	8.3 (7.5–9.0)	8.3 (7.6–9.1)	0.30
suPAR (ng/ml)	4.1 (2.9–6.0)	n.a.	
**OUTCOMES**			
Discharged within 24 hours, no. (%)	3,966 (50.2)	4,317 (48.6)	0.04*
Length of stay (days), mean (SD)	4.3 (7.4)	4.6 (9.4)	0.04*
30-day mortality	315 (4.0)	363 (4.1)	0.78
Readmissions within 30 days, no. (%)	839 (10.6)	785 (8.8)	<0.001*

CRP: C-reactive protein, IQR: Interquartile range, SD: standard deviation, suPAR: soluble urokinase plasminogen activator receptor. * P<0.05

Table S2. Patient characteristics. Comparison of patients admitted in control periods and patients arriving in interventional periods, but who did not have a valid suPAR level at admission.

Characteristic	No suPAR (N=1,002)	Control group (N=7,898)	P
**Hospital, no. of patients (%)**			
Bispebjerg Hospital	562 (56.1)	3,563 (45.1)	
Herlev Hospital	440 (43.9)	4,331 (54.9)	
**Patients**			
Female sex, no. (%)	521 (52.0)	4,170 (52.8)	0.64
Age, years, mean (SD)	60.9 (20.7)	59.6 (21.4)	0.08
Charlson score, mean (SD)	0.6 (1.2)	0.7 (1.5)	0.002*
**Biomarker blood levels, median (IQR)**			
Albumin (g/L)	38 (34–42)	39 (34–42)	0.02*
Creatinine (µmol/L)	77 (64–94)	75 (62–93)	0.07
CRP (mg/L)	4 (3–28)	5 (3–41)	<0.001*
Hemoglobin (µmol/L)	8.3 (7.6–9.1)	8.3 (7.6–9.1)	0.57
**OUTCOMES**			
Discharged within 24 hours, no. (%)	463 (46.2)	3,854 (48.8)	0.12
30-day mortality	44 (4.4)	319 (4.0)	0.66
Length of stay (days), mean (SD)	5.0 (13.5)	4.5 (8.7)	0.29
30-day mortality	44 (4.4)	319 (4.0)	0.66
Readmissions within 30 days, no. (%)	78 (7.8)	707 (9.0)	0.24

CRP: C-reactive protein, IQR: Interquartile range, SD: standard deviation, suPAR: soluble urokinase plasminogen activator receptor. * P<0.05

Table S3. Patient characteristics at index admission for patients discharged from the emergency department within 24 hours compared to patients with longer admissions

Characteristics	Discharged within 24 hours (N = 8,283)	Not discharged within 24 hours (N=8,492)	P
**Hospital, no. patients (%)**			
Bispebjerg Hospital	2,966 (35.8%)	4,049 (47.7%)	
Herlev Hospital	5,317 (64.2%)	4,443 (52.3%)	
**Patients**			
Female sex, no. (%)	4,431 (53.5%)	4,421 (52.1%)	0.07
Age, years, mean (SD)	53.5 (20.6)	67.5 (18.4)	<0.001*
Charlson score, mean (SD)	0.48 (1.2)	0.91 (1.7)	<0.001*
**Biomarker blood level, median (IQR)**			
Albumin (g/L)	41 (37–44)	37 (32–41)	<0.001*
Creatinine (μmol/L)	73 (61–87)	79 (64–102)	<0.001*
CRP (mg/L)	3.0 (3.0–14.0)	14.5 (3.0–75.1)	<0.001*
Hemoglobin (mmol/L)	8.6 (7.9–9.2)	8.1 (7.2–8.9)	<0.001*
suPAR (ng/mL)	3.5 (3.5–4.8)	4.9 (3.5–7.2)	<0.001*

CRP: C-reactive protein, IQR: Interquartile range, SD: standard deviation, suPAR: soluble urokinase plasminogen activator receptor. P<0.05

Table S4. Patient characteristics and outcomes stratified according to hospital

Characteristic	suPAR Group	Control Group	P
**Bispebjerg Hospital: 7,020 patients**			
Allocation	2,895 (41.2)	4,125 (58.8)	
Female sex, no. (%)	1,497 (51.7)	2,198 (53.3)	0.20
Age, years, mean (SD)	59.6 (21.4)	60.4 (21.0)	0.11
Discharged within 24 hours, no. (%)	1,222 (42.2)	1,744 (42.3)	0.96
Length of stay (days), mean (SD)	5.0 (8.4)	5.1 (10.6)	0.70
30-day mortality, no. (%)	119 (4.1)	176 (4.3)	0.76
Readmissions within 30 days, no. (%)	300 (10.4)	385 (9.3)	0.15
**Herlev Hospital: 9,781 patients**			
Allocation	5,010 (51.2)	4,771 (48.8)	
Female sex, no. (%)	2,676 (53.4)	2,493 (52.3)	0.26
Age, years, mean (SD)	61.0 (20.2)	61.1 (20.6)	0.98
Discharged within 24 hours, no. (%)	2,744 (54.8)	2,573 (53.9)	0.48
Length of stay (days), mean (SD)	3.9 (6.6)	4.1 (8.2)	0.15
30-day mortality, no. (%)	196 (3.9)	187 (3.9)	1
Readmissions within 30 days, no. (%)	539 (10.8)	400 (8.4)	<0.01

SUPPLEMENTARY FIGURES

Figure S1. Survival of patients discharged within 24 hours from the emergency department stratified according to suPAR quartiles measured at index admission. Log rank test: P<0.001.

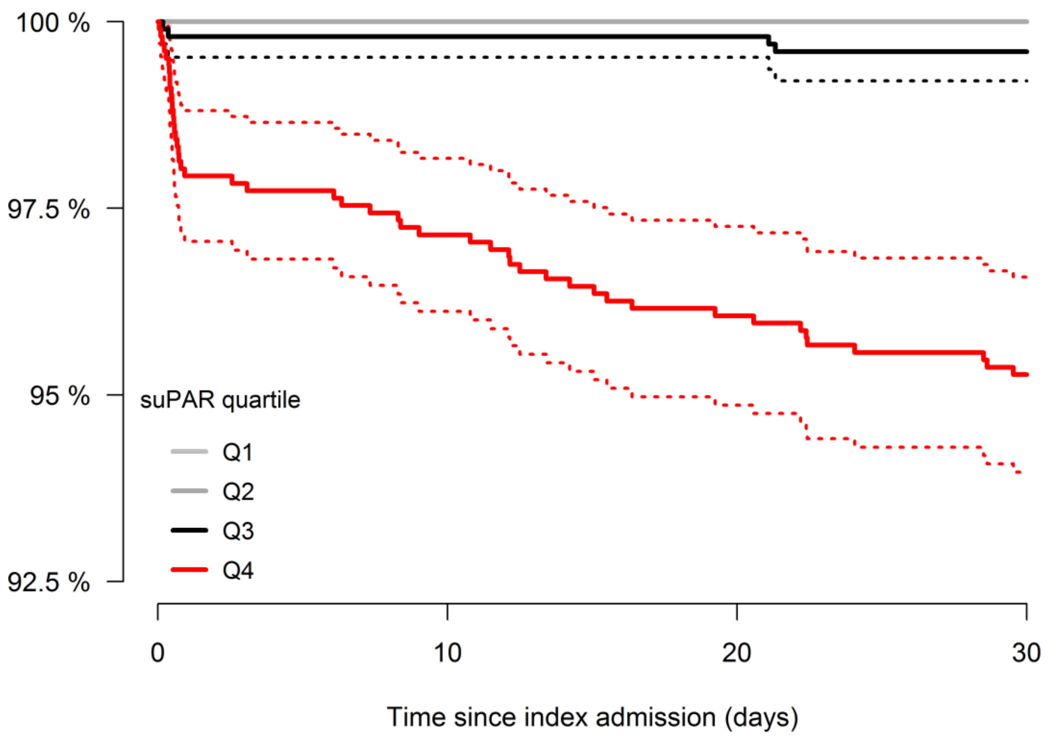

suPAR quartile Patients at risk				
Q1: <2.6 ng/mL	905	905	905	905
Q2: 2.6-3.5 ng/mL	1048	1048	1048	1048
Q3: 3.5-4.8 ng/mL	998	996	995	993
Q4. >4.8 ng/mL	1015	986	975	967

Figure S2 ROC curve comparison of biomarker model and suPAR. Receiver operating characteristics curves for 30-day all-cause mortality displaying predictive abilities of a biomarker model (including levels of albumin, creatinine, C-reactive protein, and haemoglobin), the biomarker soluble urokinase plasminogen activator receptor (suPAR), as well as the biomarker model combined with suPAR. No suPAR: biomarker model without suPAR; suPAR included: biomarker model including suPAR.

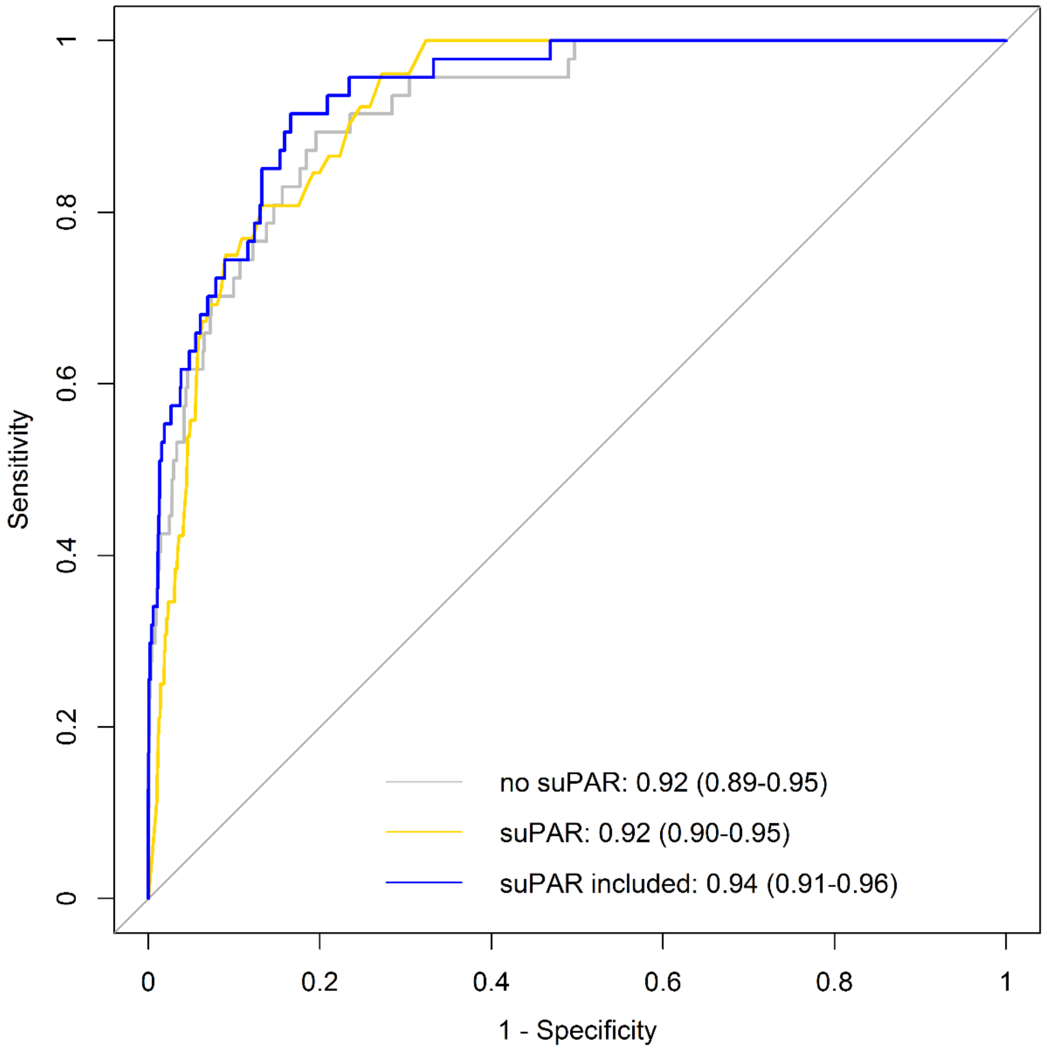

Supplement: Supplementary Materials — The file contains Tables S1, S2, S3, and S4 and Figures S1 and S2. These supplementing tables and figures provide a more in-depth understanding of the manuscript. Table S1: patient characteristics and outcomes. Groups were created based on whether suPAR was measured and reported at the index admission (suPAR group) or not (control group). Table S2: patient characteristics. Comparison of patients admitted in control periods and patients arriving in interventional periods, but who did not have a valid suPAR level at admission. Table S3: patient characteristics at index admission for patients discharged from the emergency department within 24 hours compared to patients with longer admissions. Table S4: patient characteristics and outcomes stratified according to hospital. Figure S1: survival of patients discharged within 24 hours from the emergency department stratified according to suPAR quartiles measured at index admission. Log-rank test: P < 0.001. Figure S2: ROC curve comparison of a biomarker model and suPAR. Receiver operating characteristic curves for 30-day all-cause mortality displaying predictive abilities of a biomarker model (including levels of albumin, creatinine, C-reactive protein, and haemoglobin), the biomarker soluble urokinase plasminogen activator receptor (suPAR), and the biomarker model combined with suPAR. No suPAR: a biomarker model without suPAR; suPAR included: a biomarker model including suPAR. [file 3403549.f1.pdf]
